# Mangiferin Against Respiratory Diseases: Pharmacological Targets and Prospects

**DOI:** 10.1002/prp2.70163

**Published:** 2025-08-10

**Authors:** Kazi Ahsan Ahmed, Nusrat Afrin, Popy Ghosh, Irin Amin Heya, Sajidur Rahman Akash, Akhi Moni, Mohammad Nazrul Islam, Md. Golzar Hossain, Alessandra Sinopoli, Md Abdul Hannan, Md Jamal Uddin

**Affiliations:** ^1^ ABEx Bio‐Research Center Dhaka Bangladesh; ^2^ Department of Biochemistry and Molecular Biology Bangladesh Agricultural University Mymensingh Bangladesh; ^3^ Department of Biotechnology Sher‐e‐Bangla Agricultural University Dhaka Bangladesh; ^4^ Department of Microbiology and Hygiene Bangladesh Agricultural University Mymensingh Bangladesh; ^5^ Department of Prevention Local Health Authority Roma 1 Rome Italy

**Keywords:** fibrosis, infections, inflammation, Mangiferin, oxidative stress, respiratory diseases

## Abstract

Respiratory diseases are associated with high mortality worldwide. Respiratory infections can lead to the emergence of chronic respiratory diseases. Scientists are constantly striving to identify new therapies with reduced side effects. The rise of antibiotic resistance and the scarcity of effective treatments further necessitate the development of novel therapeutics specific to respiratory diseases. Extensive research has been conducted on natural products that could be effective against respiratory diseases. Mangiferin, a polyphenol with a C‐glycosyl xanthone structure, is a bioactive phytochemical that has potential applications in the treatment of respiratory tract infections. Mangiferin could be a therapeutic option against respiratory diseases because of its ability to target a variety of pharmacological pathways implicated in the development of these infections. It has been shown to limit infections, lower inflammation, control immune responses, and enhance host defense mechanisms. This review provides comprehensive insight into mangiferin's potential against various respiratory disorders, focusing on its pharmacological activity and therapeutic prospects. Despite the potential of mangiferin against respiratory problems‐related pathobiology, additional scientific validation through clinical trials is required before the clinical application of mangiferin in the management of respiratory diseases.

## Graphical abstract

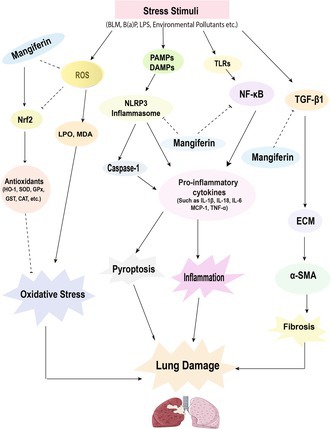

## 1 Introduction

Respiratory diseases are linked to a high mortality rate worldwide [1]. Seasonal epidemics of the common cold and influenza have long been well‐known indicators of respiratory viral disorders [2]. Respiratory viruses are a persistent reason for concern across all population subgroups [3]. Respiratory infections are a major cause of diseases and casualties worldwide [4]. There have been an increasing number of respiratory viruses, including coronavirus, influenza viruses, MERS viruses, adenovirus, metapneumovirus, respiratory syncytial virus, and rhinovirus, that might cause symptoms such as disseminated alveolar destruction and acute respiratory failure, rapidly evolving into acute respiratory distress syndrome (ARDS) [5, 6, 7].

Numerous respiratory infectious diseases exist for which novel therapeutic approaches are required. Respiratory viruses that replicate in the epithelial lining of the airways also contribute to inflammation [8, 9]. In addition, a common set of chronic respiratory illnesses is caused by various bacteria [10]. Chronic obstructive pulmonary disease (COPD) is influenced by bacterial invasion and persistence in respiratory tissues, which alter the host response and induce a chronic inflammatory response. Bacteria are mostly responsible for chronic bronchitis, pathogen colonization of the lower respiratory tract, and progressive airway blockage [11, 12]. COPD, which largely occurs in respiratory inflammation, is the fifth most frequent cause of mortality in the world [13, 14]. The disease condition may affect both the upper and lower airways and worsen conditions such as rhinosinusitis [15]. ARDS may cause worse outcomes, especially for elderly people and people who have comorbid conditions like asthma, diabetes mellitus, or cardiovascular disease [7]. Thus, effective treatments against respiratory disease are urgently needed.

**FIGURE 1:**
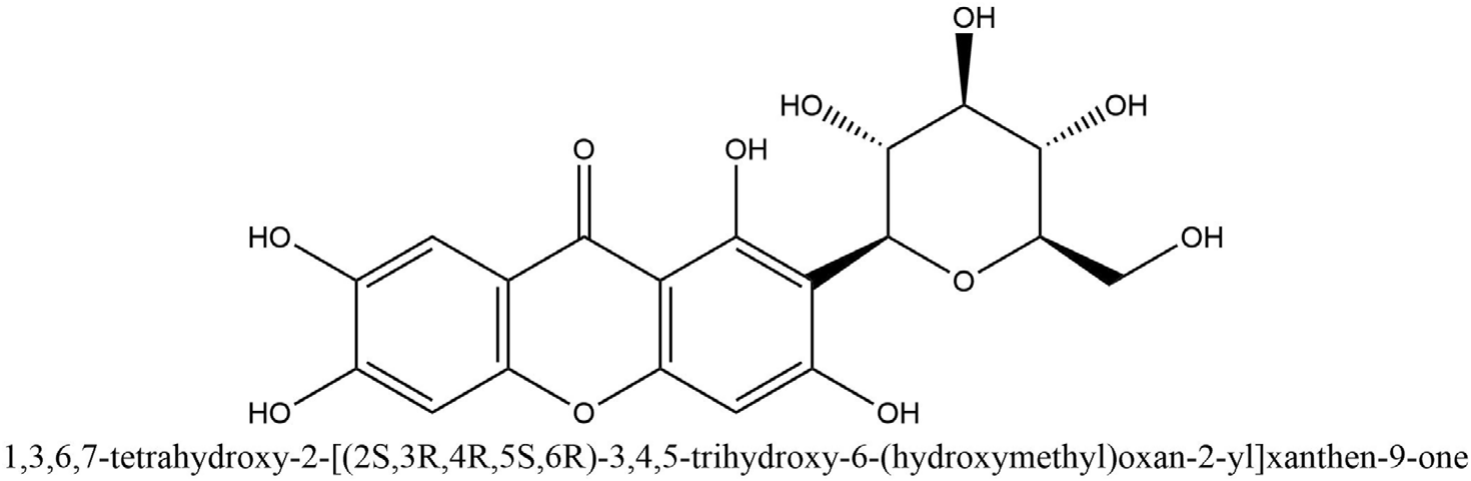
Structure of mangiferin (PubChem CID 5281647).

Various types of phytochemicals including alkaloids, flavonoids, glycosides, lignans, polyphenols, and saponins are being investigated for treatments of respiratory diseases [8]. A number of traditional plants, notably *Mangifera indica* L., *Anemarrhena asphodeloides*, *Belamcanda chinensis* (L.) DC., *Salacia hainanensis, and Mangifera persiciformis*, contain mangiferin as one of the major active ingredients [16, 17]. Mangiferin (1,3,6,7‐tetrahydroxyxanthone‐C2‐β‐D‐glucoside) (Figure 1) is a naturally occurring glucoxilxanthone that may be obtained from various parts of the mango plant [18, 19].

Mangiferin is a promising option for the treatment and prevention of respiratory disorders due to its various pharmacological activities, which include antidiabetic, cardioprotective, neuroprotective, antioxidant, anti‐inflammatory, antipyretic, analgesic, antibacterial, antiviral, renoprotective, anticancer, and immunomodulatory effects [18, 20, 21, 22, 23, 24, 25, 26, 27]. Mangiferin has shown potential and benefits in animal models of respiratory syndrome [8]. Mangiferin has also been shown to improve antioxidant, anti‐inflammatory, and antifibrotic effects in respiratory tissues in many studies [28]. It appears promising as a remedy for allergic rhinitis, lessens lung inflammation, and reduces the risk of asthma [29]. Mangiferin reduces reactive oxygen species (ROS) levels in respiratory tissues and may enhance antioxidant activity [30]. It also has promising effects on respiratory disorders by modulating major cytokines and signaling pathways by lowering inflammation and tissue fibrosis [31, 32, 33, 34]. Despite its potential, mangiferin has a few drawbacks, including low bioavailability and minor side effects such as dyspnea, flank position, and piloerection shown in animal models [35, 36]. Several studies suggest innovative delivery methods and combination therapies may improve its effectiveness [37]. Although mangiferin shows potential benefits in the treatment of respiratory diseases, there have been no substantial clinical trials to confirm these preclinical results for patient use. A comprehensive literature review is thus timely and may help identify additional research that could enable the development of this natural product for the management of respiratory diseases. In this narrative literature review, various online databases were explored for existing reports on the effectiveness of mangiferin in treating respiratory issues with an emphasis on molecular pharmacology.

## 2 Methodology

**FIGURE 2:**
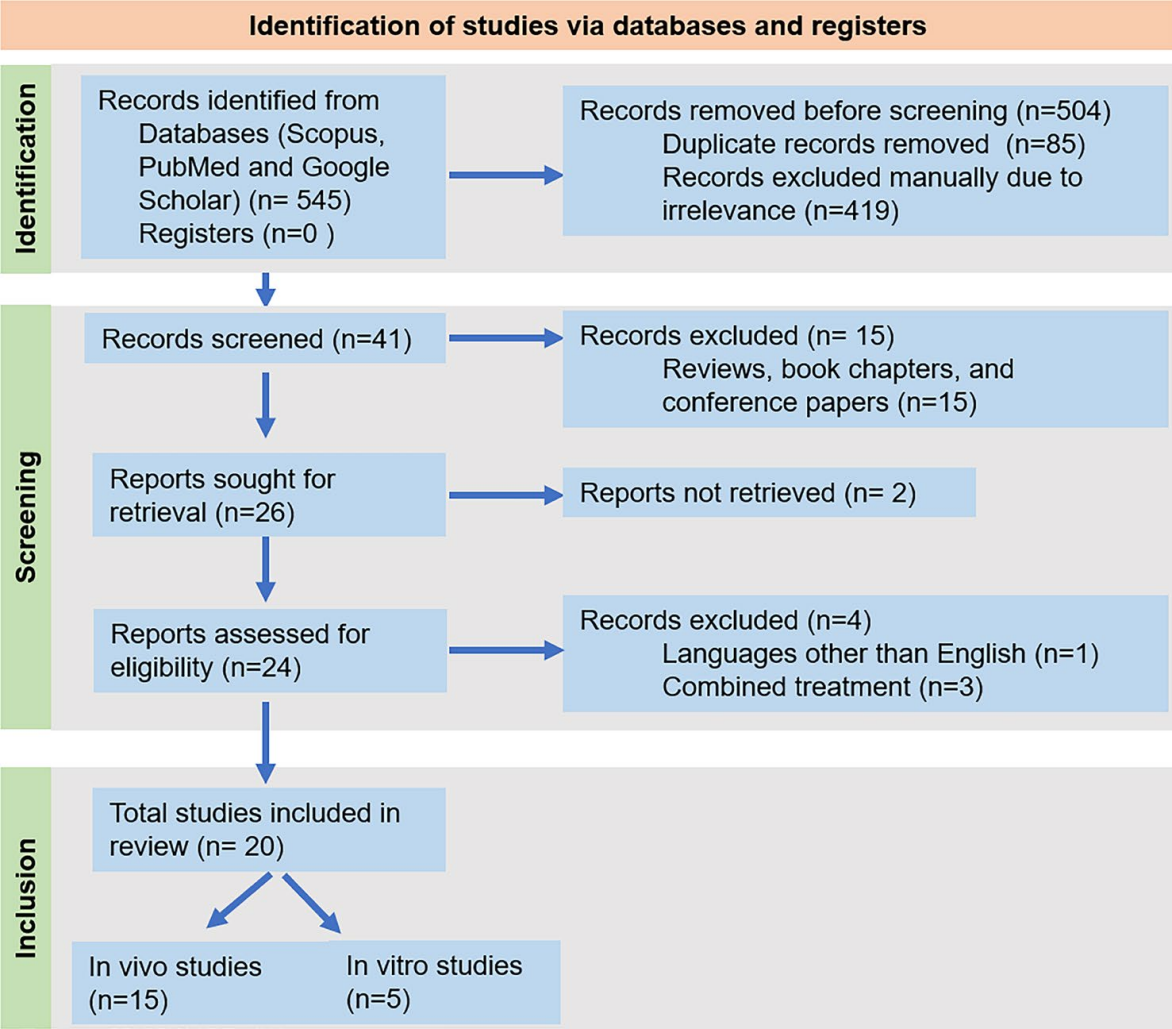
PRISMA 2020 flow diagram for the systematic review of mangiferin effects against respiratory diseases.

The literature being reviewed was identified, screened and finally included systematically following the method of Preferred Reporting Items for Systematic Reviews and Meta‐Analysis (PRISMA) (Figure 2). Several scientific databases and homepages, including Web of Science, PubMed, and Google Scholar were accessed for various published data, reviews, and research findings on the effectiveness of mangiferin in preventing respiratory tract infections. The search keywords included “respiratory infection”, “bioactive compound” and “mangiferin on respiratory diseases or lung diseases” in all fields. The data was obtained between 1996 and January 2022. ChemDraw Professional 16.0 and Adobe Illustrator were used to create the figures. Insights from these studies, taken in a larger context, may be used to treat lung and pulmonary infections as well as other respiratory tract infections.

## 3 Pathophysiology of Respiratory Diseases

**FIGURE 3:**
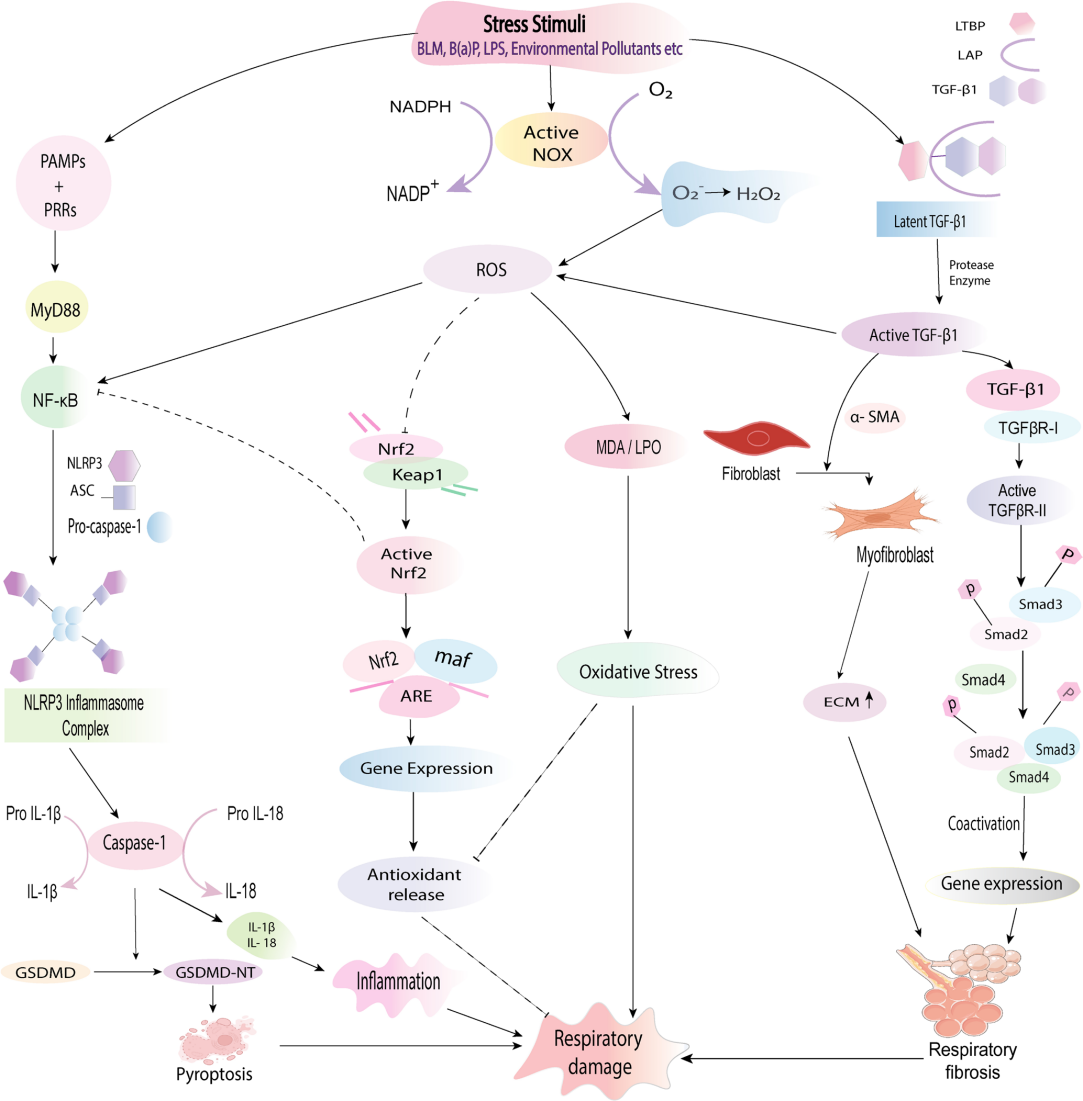
Signaling pathways involved in the pathogenesis of respiratory damage. Under stress conditions (e.g., BLM, B(a)P, environmental pollutants), the respiratory cell can be damaged by immune‐mediated signaling pathways. NOX favors the conversion of NADPH to NADP+ by forming superoxide free radicals which in turn produce H_2_O_2_. Upregulated ROS causes MDA production that induces respiratory cell damage. Association of PRRs with PAMPs results in MYD88 and further NF‐κB stimulation, leading to the expression of the NLRP3 inflammasome complex. The NLRP3 inflammasome then induces caspase‐1 to convert pro‐IL‐1β, pro‐IL‐18, and GSDMD for producing IL‐1β, IL‐18, and GSDMD‐NT in a respective manner. As a result, pyroptosis occurs. Following cleavage of latent TGF‐β1, active TGF‐β1 binds with TGFβR‐II and activates TGFβR‐I, leading to the expression of phosphorylated Smad2 and Smad3. Following the attachment with Smad4 and in the presence of coactivators, gene expression occurs that incites respiratory fibrosis. Through the use of α‐SMA, TGF‐β1 creates myofibroblasts that cause elevated ECM and ultimately fibrosis. Active TGF‐β1 influences ROS generation, and redox imbalance in turn affects TGF‐β1 production. Redox imbalance further impels fibrosis. Nrf2 dissociates from Keap1 via ROS and helps in gene expression that generates antioxidants for suppressing cell damage. Nrf2 can inhibit cell damage by suppressing NF‐κB (induced by ROS) signaling that causes altered pro‐inflammatory cytokine levels and inflammasome complex inhibition.

The main pathophysiological features of respiratory diseases, including inflammatory response, oxidative stress, fibrosis, and other related pathologies, are described in this section (Figure 3).

Oxidative stress is an imbalance between the production and accumulation of reactive oxygen species (ROS) and antioxidant enzymes in cells, leading to a pathological condition [38, 39, 40]. Generally, immune cells produce ROS in response to internalized stress as part of the immune defense mechanism [38]. ROS involved in the immune response is produced via NADPH oxidase (NOX) [41]. NOX is made up of cytosolic p47phox, p40phox, p67phox, and p21 Rac subunits and transmembrane gp91phox and p22phox subunits. When these regulatory subunits come together with gp91phox, NOX becomes activated. Following assembly and activation, NOX facilitates the generation of a superoxide free radical by transferring an electron from NADPH to molecular oxygen and forming NADP^+^. However, the extremely reactive “non‐diffusible” superoxide ion undergoes spontaneous or enzymatic conversion to generate H_2_O_2_, which is detrimental to the cells [38, 42]. Malondialdehyde (MDA) is generated as a result of the peroxidation of polyunsaturated fatty acids within cells. An excessive accumulation of free radicals leads to an elevated production of MDA that disrupts the integrity and functionality of the cell membrane [43, 44].

Inflammation is one type of essential defense mechanism of the immune system of the body against harmful agents and tissue damage, aiming to remove them and promote healing. While it is crucial for health, uncontrolled inflammation can play a role in various diseases [45, 46, 47]. Different pathways of inflammatory response can cause cell damage (Figure 3). Pathogen recognition receptors (PRRs) of the immune system serve as the primary detectors that identify specific patterns known as PAMPs found on microbes [48]. Upon detection, these PRRs can trigger an inflammatory response by promoting the release of pro‐inflammatory molecules through subsequent signaling pathways that contribute to inflammation [49, 50]. There are four types of PRRs, consisting of TLRs, RLRs, NLRs, and CLRs [49, 51]. These receptors activate a series of signaling pathways, including NF‐κB, MAPK, and JNK, where cytokines, interferons, and chemokines are synthesized as a result that helps to neutralize stress by cells like neutrophils, eosinophils, monocytes, natural killer cells, macrophages, and dendritic cells [51, 52, 53].

Inflammasomes are complex assemblies of multiple proteins consisting of specific PRRs that can identify PAMPs. NOD‐like receptor family pyrin domain containing 3 (NLRP3) is one type of NLR receptor as well as an inflammasome [51, 54]. After the detection of PAMPs, it promotes the stimulation of MYD88 and activation of NF‐κB, which induce the expression of three key components of the inflammasome complex (NLRP3 protein, ASC, and pro‐caspase‐1), leading to the release of pro‐interleukin‐1β and pro‐IL‐18 [55, 56, 57]. Functional NLRP3 inflammasome influences caspase‐1 that cleaves pro‐IL‐1β and pro‐IL‐18 to produce mature IL‐1β and IL‐18 that contribute to inflammation and respiratory damage [51, 55, 57, 58]. In pulmonary fibrosis, IL‐1β drives inflammation by attracting neutrophils and lymphocytes, and promoting collagen synthesis, while IL‐18 triggers epithelial‐mesenchymal transition (EMT) and lung fibroblast senescence by reducing the anti‐senescence protein Klotho [59]. In the presence of excessive levels of ROS, Nrf2 is released from Keap1. Nrf2 binds with Maf protein and ARE, leading to gene expression that produces antioxidants and inhibits cell damage. Additionally, Nrf2 restrains NF‐κB activation, resulting in downregulated pro‐inflammatory cytokine levels that ultimately protect against cell damage. NF‐κB is mainly triggered by ROS through IκB phosphorylation and aids in the formation of inflammasome complexes [60, 61, 62]. Furthermore, studies have shown that under conditions of elevated mROS and H_2_O_2_, TXNIP is released from TRX. TXNIP then interacts with NLRP3 and assists in the formation of the NLRP3 inflammasome complex [55].

In respiratory fibrosis, TGF‐β1 is one type of pleiotropic cytokine that plays both a protective role by regulating immunity and a pathogenic role by promoting fibrosis [63, 64]. TGF‐β1 is kept dormant by the complex involving latent TGF‐β1, LAP, and LTBP [65]. The active TGF‐β1 homodimer can be liberated from the latent TGF‐β1 complex by a breakdown via protease [66]. Upon activation, TGF‐β1 creates an attachment with type II receptor (TGFβR‐II) that causes stimulation of TGFβR‐I. The Smad2 and Smad3 proteins are phosphorylated as a result. Then, phosphorylated Smad2 and Smad3 link up with Smad4 [67]. Along with other transcription factors as coactivators, the excited Smad complexes play a crucial role in gene expression and secrete ECM protein, collagen, profibrotic mediators, and other molecules [68]. Pulmonary fibrosis is one type of long‐lasting progressive respiratory condition characterized by ongoing cycles of tissue damage and healing. These repetitive processes result in permanent changes to the lung structure as well as tissue stiffening [69]. Fibrotic scars are featured by the buildup of matrix proteins that have contractile properties [70]. In the presence of α‐SMA, TGF‐β1 converts fibroblasts to myofibroblasts which produce ECM and cause fibrosis [69]. Moreover, TGF‐β1 induces ROS generation through stimulating NOX, reducing antioxidant activity and defecting mitochondria. In turn, ROS affects various pathways of TGF‐β1 such as the Smad pathway. The dysregulation between TGF‐β1 and ROS influences the fibrogenic effects of TGF‐β1. A redox imbalance, driven by upregulated ROS or downregulated antioxidants, promotes the activation of latent TGF‐β1 and prompts the expression of the TGF‐β1 gene, ultimately resulting in higher TGF‐β1 activity [67].

Pyroptosis, a form of cellular death triggered by proinflammatory signals, is associated with inflammatory responses. Caspase‐1 cleaves GSDMD, producing GSDMD‐NT as an N‐terminal cleavage product, which forms membrane pores, leading to pyroptosis due to its affinity for plasma membrane lipids [71] (Figure 3).

## 4 Pharmacological Potentials of Mangiferin Against Respiratory Diseases

**FIGURE 4:**
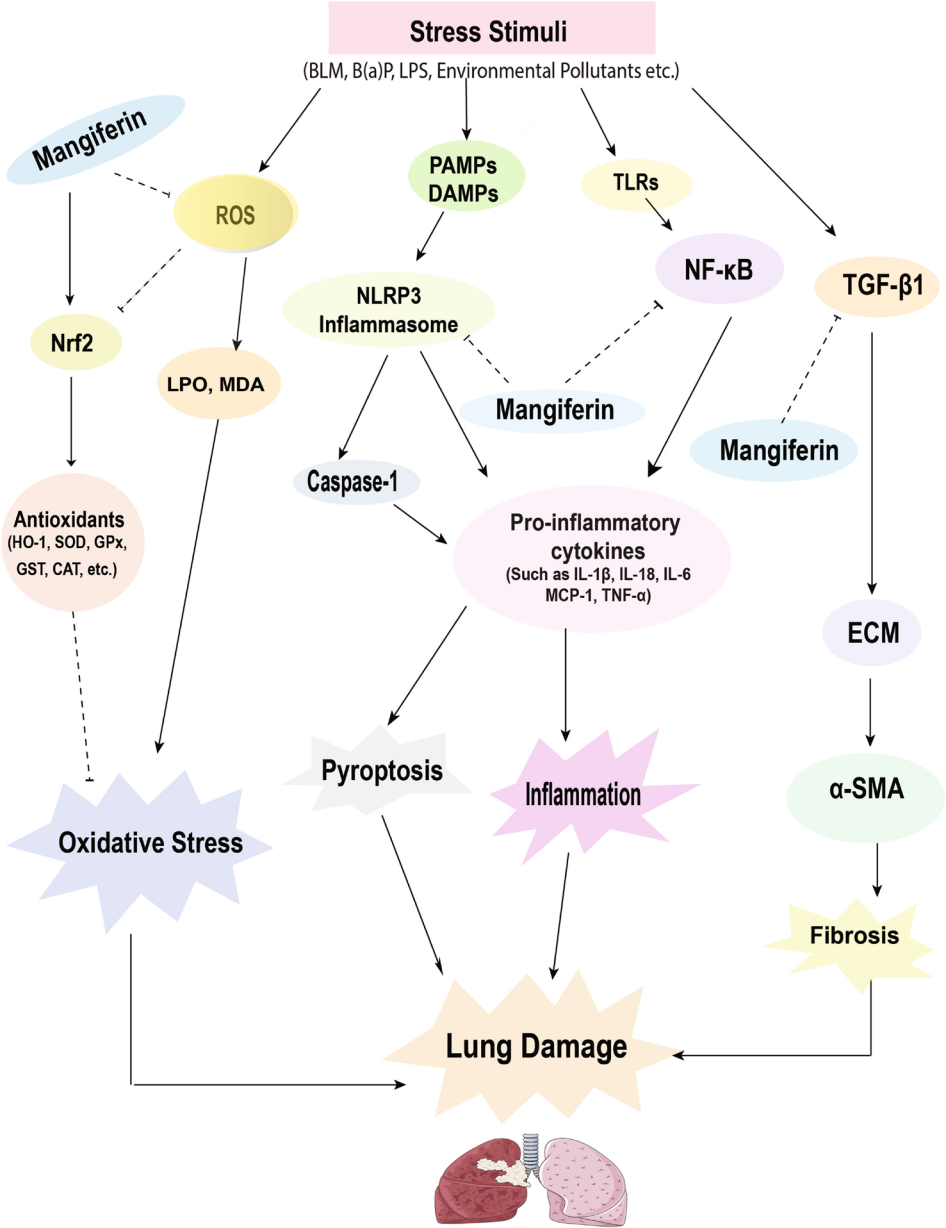
Effects of mangiferin on respiratory tract infection. The diagram represents a variety of stress stimuli, for example, Bleomycin, LPS, B(a)P, environmental pollutants, etc. that mediate pyroptosis, inflammation, and oxidative stress, which ultimately lead to lung damage. Stress stimuli activate the NLRP3 inflammasome and NF‐κB pathway, which triggers tumor necrosis factor‐α (TNF‐α), interleukin (IL)‐1β, IL‐6, IL‐18, and MCP‐1, causing inflammation and eventually lung damage, and mangiferin acts by inhibiting NF‐κB and activation of the NLRP3 inflammasome. Mangiferin also activates the Nrf2/HO‐1 pathway and produces antioxidant enzymes like HO‐1, SOD, GPx, GST, CAT, GR, QR, and inhibits ROS production, eventually LPO, MDA release, thus inhibiting oxidative stress. In addition, TGF‐β1 causes accumulation of ECM and expressing α‐SMA. Thus, the lungs become protected by mangiferin, which is known to suppress the cascades of fibrosis.

Figure 4, Tables 1 and 2 illustrate the pharmacological potentials of mangiferin against a number of pathophysiological mechanisms (i.e., oxidative stress, inflammation, fibrosis, and other pathologies) responsible for respiratory infection.

**TABLE 1:** Protective effects of mangiferin against respiratory problems in vivo.

Model animals	Disease inducing agents	Mangiferin dosage			Effects of mangiferin				Ref.
		Dose	Route	Duration	Oxidative stress	Inflammation	Fibrosis	Other pathologies	
Mice	BLM	10 mg/kg	Orally	7 days	↓ROS	↓TNF‐α, IL‐1β, iNOS, Nitro tyrosine, and COX‐2	_	↓ PARP, Bid, NF‐κB p65, and MPO, ↑Bcl‐2	[31]
Mice	BLM	40 mg/kg	Orally	14 days	↓ROS	↓TNF‐α, IL‐6, MCP‐1, and IL‐1β	↑TGF‐β1, α‐SMA	↓TLR4/NF‐κB pathway	[32]
Mice	Ovalbumin	50 mg/kg,100 mg/kg,200 mg/kg	Orally	14 days	_	↑IL‐2, IL‐10, IL‐12, and IFN‐γ, ↓IL‐3, IL‐4, IL‐5, IL‐9, IL‐13, IL‐17 RANTES, and TNF‐α.	_	↓STAT6 signaling pathway	[72]
Mice	Ovalbumin	5 mg/kg, 20 mg/kg	Orally	13 days	↓MDA, and ROS, ↑SOD	↑IL‐12, IFN‐γ, ↓IL‐4, IL‐5, IL‐13, IL‐17, TNF‐α, and NF‐κB	_	↓GATA‐3, and RORγ, ↑Nrf2/HO‐1 pathway and T‐bet	[73]
Mice	Ovalbumin	50 mg/kg	Orally	24 days	_	↓IL‐4 and IL‐5	_	↓IgE	[29]
Mice	Ovalbumin	100 mg/kg, 200 mg/kg	Orally	14 days	_	↓IL‐9, IL‐17A, ↑IL‐10	_	↓PU.1, and RORγt, ↑Foxp3	[74]
Mice	LPS	100 mg/kg	Orally	1 h to 7 h	_	↓IL‐1β, IL‐18, and NLRP3	_	_	[75]
Mice	LPS	20 mg/kg, 50 mg/kg, 100 mg/kg	Orally	7 days	↓ROS, and MDA, ↑SOD	↓HMGB1 and NF‐κB	_	_	[28]
Mice	B(a)P	100 mg/kg	Orally	18 weeks	↑SOD, and CAT, ↓LPO	_	_	↑ IgG and IgM, ↓IgA	[76]
Mice	B(a)P	100 mg/kg	Orally	18 weeks	↓LPO, ↑GST, QR, and UDP‐GT	_	_	↓CYP, Cytb_5_, NADPH and Cyt c	[77]
Mice	B(a)P	100 mg/kg	Orally	18 weeks	↓ROS, ↑SOD, GSH, GPx, GR, and GST	_	_	_	[78]
Mice	CLP	10 mg/kg, 30 mg/kg, 100 mg/kg	Orally	7 days	_	↑TNF‐α, IL‐6, HO‐1 ↓COX‐2, iNOS	_	↓MAPK and NF‐κB	[79]
Mice	CLP	2.5 mg/mL, 5 mg/mL	Orally	2 days	_	_	_	↓VCAM‐1	[80]
Rats	Cigarette Smoke	10 mg/kg, 100 mg/kg	IG	6 days	↓ROS, and MDA, ↑SOD	↑HO‐1	_	↑Nrf‐2 signaling, and 𝛾‐GCS	[30]
Mice	Arsenic	40 mg/kg	Orally	5 Weeks	↓ROS, LPO, and MDA	↓MPO, TNF‐α, IL‐1β, IL‐6, MCP‐1, VCAM‐1, ICAM‐1, and VEGF	_	↓NF‐κB, Caspase 3, 8, 9, and Bax, ↑Bcl2	[81]

Abbreviations: α‐SMA, α‐smooth muscle actin; 𝛾‐GCS, γ‐glutamylcysteine synthetase; B(a)P, Benzo(a)Pyrene; Bax, Bcl‐2 associated X protein; Bcl‐2, B‐cell lymphoma 2; BLM, bleomycin; CAT, catalase; CLP, cecal ligation and puncture; COX‐2, cyclo‐oxygenase‐2; CYP, cytochrome P450; Cytb_5_, microsomal cytochrome b_5_; Cyt c, cytochrome complex; DAMPs, damage‐associated molecular patterns; ECM, extracellular matrix; Foxp3, Forkhead Box P3; GATA‐3, GATA binding protein 3; GPx, glutathione peroxidase; GR, glutathione reductase; GSH, glutathione; GST, glutathione S‐transferase; HMGB1, high mobility group box 1; HO‐1, heme oxygenase‐1; ICAM‐1, Intercellular adhesion molecule 1; IFN‐γ, interferon gamma; Ig, immunoglobulins; IG, intragastrically; IL, interleukins; iNOS, inducible NO synthase; LPO, lipid peroxidation; LPS, lipopolysaccharide; MAPK, mitogen‐activated protein kinase; MCP‐1, monocyte chemoattractant protein 1; MDA, malondialdehyde; MPO, myeloperoxidase; NADPH, nicotinamide adenine dinucleotide phosphate; NF‐κB, nuclear factor kappa B; NLRP3, nucleotide‐binding domain, leucine‐rich‐containing family, pyrin domain‐containing protein 3; Nrf2, nuclear factor erythroid 2; PAMPs, pathogen‐associated molecular patterns; PARP, poly (ADP‐ribose) polymerase; PU.1, tissue‐specific transcription factor PU.1; QR, quinone reductase; RANTES, regulated upon activation; ROR, RAR‐related orphan receptor; ROS, reactive oxygen species; SOD, superoxide dismutase; STAT6, signal transducer and activator of transcription 6; T‐bet, T‐box expressed in T cells; TGF‐β1, transforming growth factor‐β1; TLR4, toll‐Like Receptor 4; TLRs, toll‐like receptors; TNF‐α, tumor necrosis factor‐α; UDP‐GT, uridine diphosphate glucoronyltransferase; VCAM‐1, vascular cell adhesion molecule 1; VEGF, vascular endothelial growth factor.

**TABLE 2:** Protective effects of mangiferin against respiratory problems in vitro.

Cell lines	Model drug	Mangiferin		Effects of mangiferin			Ref.
		Dose	Duration	Oxidative stress	Inflammation	Other pathologies	
NSCLC cell lines (A549, NCI‐H460, and NCI‐H520)	LPS	25 μg/mL and 3.125 μg/mL	4,24, and 48 h	—	↓CXCR4 TNF‐α, IL‐6, and IL‐1β ↓NLRP3	↓NSCLC cell proliferation ↑Caspase‐8 ↑autophagic LC3β/LC3α ↑E‐cadherin ↓Vimentin	[82]
BEAS‐2B cells	PAH	0.5 μg/mL	24, 48, and 72 h	ABTS•^+^ and DPPH•	—	↑bovine aortic endothelial cell migration, ↓GSK‐3β and NF‐κβ	[83]
A5A9 cells	TGF‐β1	10 μg/mL	24 h	—	—	↓EMT ↓MMP‐9 ↓p‐Smad2 and p‐Smad3	[32]
RAW264.7 macrophages and N9 microglia cell line	LPS and IFNγ	0.01 μg/mL and 2 U/mL	24 h	—	↓TNF‐α ↓NO		[84]
A5A9 cells	—	25 μg/mL	12, 24, 36 and 48 h	—	—	↓PKC/NF‐κB pathway ↓apoptosis ↓cdc2‐cyclin B1 signaling pathway (induces G2/M cell cycle arrest)	[85]
BEAS‐2B cells	TNF‐α	42.23 μg/mL	6, 24 h	—	↓NF‐κβ ↓TNF‐α, ↓IL‐1, ↓IL‐6, and ↓IL‐12 ↓PGE_2_, ↓PGD_2_		[86]

Abbreviations: A5A9, adenocarcinomic human alveolar basal epithelial cells; ABTS•^+^, 2,2′‐azino‐bis(3‐ethylbenzothiazoline‐6‐sulfonic acid); BEAS‐2B, human bronchial epithelial cell line; cdc2, cyclin‐dependent kinase 1; CXCR4, chemokine receptor type 4; DPPH, diphenyl picryl hydrazine; EMT, epithelial‐mesenchymal transition; GSK‐3β, glycogen synthase kinase—3 beta; IFNγ, interferon gamma; IL‐1β, interleukin 1 beta; IL‐6, interleukin 6; LC3β, murine light chain 3 beta; LPS, lipopolysaccharide; MMP‐9, matrix metalloproteinase −9; N9, human glial cell line; NCI‐H460, lung cancer cell line; NF‐κβ, nuclear factor kappa B; NLRP3, “NOD‐like” receptor family pyrin domain containing 3; NO, nitric oxide; NSCLC, non‐small cell lung cancer; PAH, polycyclic aromatic hydrocarbon; PER1, period circadian regulator 1; PGD_2_, Prostaglandin D2; PGE2, Prostaglandin E2; PKC, protein kinase C; pSmad2, phosphorylated–Smad2; RAW264.7, monocyte/macrophage‐like cells; TGF‐β1, transforming growth factor beta 1; TNF‐α, tumor necrosis factor alpha.

### 4.1 Effects of Mangiferin on Oxidative Stress in Lung

Oxidative stress occurs when cellular ROS levels and antioxidant enzymes become imbalanced and cause pathological conditions [40]. The generation of ROS controls several signaling pathways, such as cell division and development, mitogenesis, the synthesis and degradation of extracellular matrix (ECM), inflammation, and apoptosis. An optimal level of ROS participates normally in cellular metabolism through cell‐to‐cell interactions, but an elevated level of ROS may cause serious injury to cells and tissues [87]. The antioxidative defense mechanism of cells is important to reduce the damage that occurs due to oxidative stress. Mangiferin has many biological functions as an antioxidant and anti‐inflammatory bioactive compound [82]. By reducing the intercellular ROS, it keeps a balance between ROS and antioxidants in the respiratory tissues and eventually normalizes the activity of superoxide dismutase (SOD) reported in many experiments in mice and rats (Table 1).

By producing ROS, bleomycin (BLM) modifies the equilibrium between oxidants and antioxidants in the lungs, leading to oxidative stress [31]. Mangiferin significantly reduced the level of respiratory ROS in lung cells [31, 32]. By raising ROS in cells, a variety of medications and chemical substances are known to cause oxidative damage. The main ingredient of egg white, ovalbumin, generates MDA and ROS, which cause oxidative stress and lower SOD levels. Interestingly, mangiferin was found to increase antioxidant enzymes and reduce ROS and MDA in ovalbumin‐induced mice [29, 72, 73, 74]. Mangiferin reduces lipopolysaccharide (LPS)‐induced sepsis and recovers sepsis‐associated organ dysfunction [28].

Benzo[a]pyrene (B(a)P) is a kind of polycyclic aromatic hydrocarbon (PAH) noticed in different smoked and grilled food products, cigarette smoke, and byproducts of industrial waste incineration. The free‐radical production is one of the major mechanisms of B(a)P inducing oxidative stress in cells [88]. Interestingly, mangiferin provoked antioxidants including phase II enzymes like SOD, CAT, QR, UDP‐GT, GST, and GSH, GPx, GR as well as reduced ROS and LPO [76, 77, 78].

A variety of studies have suggested that mangiferin can target signaling pathways and reduce the adverse effects of immune response by decreasing the levels of ROS, MDA, and ameliorating the levels of different essential enzymes [18]. Furthermore, mangiferin plays a role in inducing the Nrf2 signaling pathway for protecting cells from damage [33, 34]. Also, mangiferin reduced oxidative stress in animal models where cigarette smoke and arsenic were used as ROS inducers [30, 81].

### 4.2 Effects of Mangiferin on Lung Inflammation

The goal of the biological process known as inflammation, which is triggered by damaging stimuli, is to eradicate the source of cell damage and start the healing process [45]. Environmental contaminants, as well as bacterial and viral infections, can cause lung inflammation [89]. Furthermore, neutrophils and macrophages triggered by the inflammatory response can penetrate lung tissues after acute lung injury (ALI) and release cytokines to induce local inflammation [90]. Damage‐related molecular patterns (DAMPs) and PAMPs are two examples of danger signals that can activate the innate immune signaling receptor, NLRP3. The inflammasome's core assembly, NLRP3, can be activated to cause pyroptosis and caspase 1‐mediated proteolytic activation of IL‐18 and IL‐1β [91]. Currently, BLM is an anti‐tumor medication used to treat Hodgkin's lymphoma in humans. Lung fibrosis and inflammation could be side effects of this medication if it is taken frequently. BLM administration increased ROS overproduction, which causes lipid peroxidation and DNA damage [92] which eventually causes tissue damage and activates multiple intracellular signaling pathways that produce proinflammatory cytokines, including TNF‐α, IL‐1β, IL‐6, and MCP‐1 [93]. Inducible NO synthase (iNOS) is upregulated during inflammation. However, the administration of mangiferin reduced the concentration of each of these cytokines in mouse models of inflammation [31, 32].

Research has indicated that the primary mechanism behind the pathogenesis of asthma is thought to be the disruption of the balance between Th1/Th2 cells [94]. Proinflammatory cytokines, Th2 chemokines (IL‐4, IL‐5, and IL‐13), Th1 cytokines (IFN‐γ, IL‐2, and IL‐12), chemokines (RANTES and MCP‐1), play an important role in the pathogenesis of asthma and chronic obstructive pulmonary disease [94, 95, 96]. In an asthmatic mouse model, mangiferin treatment at the dose of 200 mg/kg substantially decreased the levels of cytokines and chemokines, namely IL‐4, IL‐5, IL‐13, IL‐3, IL‐9, IL‐17, RANTES, and TNF‐ɑ in serum and lung tissues. In contrast, it showed a significant increase in the levels of IFN‐γ, IL‐2, IL‐10, and IL‐12 [72]. Interestingly, mangiferin treatment provided notable protection against the Th1/Th2/Th17 imbalance by downregulating the levels of IL‐4, ‐5, ‐13, and 17 and upregulating the levels of IFN‐γ and IL‐12. These data indicated a protective effect of mangiferin against the imbalance of Th1/Th2/Th17. During inflammation, NF‐κB is translocated to the nucleus. In OVA‐induced allergic rhinitis, mangiferin treatment greatly decreased NF‐κB activation in mice nasal and lung tissues along with nasal lavage fluid (NALF) [73]. During the sensitization and challenge phase, the administration of mangiferin decreased the immune cells' production of both Th2 cytokines [29]. Flow cytometry analysis revealed that the OVA‐induced asthmatic mouse model group had a lower proportion of Treg cells (IL‐10, TGF‐β1) and a larger proportion of Th9 (IL‐9) and Th17 (IL‐17) cells. Following 98.39% (HPLC) pure mangiferin administration with doses of 100 mg/kg and 200 mg/kg orally for 14 days, it was found that the proportions of Th9, Th17, and Treg cells were substantially restored [74].

The NLRP3 inflammasome facilitates pro‐IL‐1β and pro‐IL‐18 cleavage and maturation [97]. Pretreatment of mangiferin dramatically reduced the levels of IL‐1β and IL‐18 in the murine lung tissues, further supporting the idea that mangiferin could inhibit the activation of the NLRP3 inflammasome [75]. The high mobility group box 1 (HMGB1) binds to toll‐like receptors (TLR) and triggers NF‐κB signaling. The activation of NF‐κB signaling triggers an inflammatory storm in LPS‐treated mice, and interestingly, mangiferin was found to inhibit this signaling pathway [28]. In the mice treated with mangiferin, the CLP‐induced systemic inflammatory response was dose‐dependently improved as indicated by protein levels of TNF‐α and IL‐6. In the lungs, the protein levels of COX‐2 and iNOS in CLP‐operated mice were reduced with a dose‐dependent pretreatment of mangiferin [79].

MPO, a biomarker of inflammatory cell infiltration, is associated with the initiation of several pro‐oxidative and pro‐inflammatory reactions. Mice exposed to arsenic (As) had significantly elevated levels of MPO and inflammatory cytokines and distorted the alveoli architecture in mice lung tissue [81]. The administration of mangiferin significantly decreased the amount of MPO and the adverse histological changes. As expected, mangiferin administration reduced the amount of pro‐inflammatory cytokines and preserved the integrity of the alveolar‐capillary barrier [81]. In the lungs, mangiferin blocked NF‐κB signaling [28, 79, 81] and MAPK pathways during sepsis [79].

### 4.3 Effects of Mangiferin on Lung Fibrosis

Fibrotic lung disorders, which are characterized by a progressive loss of functional lung parenchyma and its replacement by the deposition of non‐functional connective tissue, are thought to be caused by injury to the alveolar epithelium and repeated attempts at healing [98, 99].

Numerous factors, including viral infections, radiation exposure, chemotherapy, chemotherapeutic drug use, and aerosolized environmental toxins, can accelerate the progression of fibrotic illness [100, 101, 102, 103]. Moreover, inherited genetic disorders, mismatches in human leukocyte antigen in transplants, myocardial infarction, obesity, diabetes, hypertension [104] and in people having long‐term inflammatory conditions like GERD, scleroderma, and rheumatoid arthritis may also contribute to its progression [105, 106, 107]. All of these factors trigger a chain reaction of coagulation and antifibrinolysis that results in clotting and the creation of a transient ECM [108]. Persistent accumulation of ECM in the lung tissue causes lung fibrosis. In consequence, platelet aggregation and the subsequent degranulation increase blood vessel permeability, which makes it easier for inflammatory cells like neutrophils and macrophages to enter the injury site. Myofibroblasts further promote wound contraction during the phase of wound formation and remodeling. When the repair process becomes disorganized, fibrosis occurs. Thus, multiple stages in the healing process can go wrong and trigger the formation of scars, which may explain the complexity of pulmonary fibrosis [109]. In a study of bleomycin (BLM)‐induced pulmonary fibrosis in mice, administration of mangiferin (40 mg/kg) exhibited potential anti‐fibrotic effects by blocking Epithelial‐Mesenchymal Transition (EMT) primarily through inhibition of Smad2/3 phosphorylation and matrix metalloproteinase‐9 (MMP‐9) [32].

The antifibrotic medications pirfenidone [110, 111] and nintedanib [112, 113] have demonstrated their efficacy and safety in phase II and III clinical trials. TGF‐β1 plays a well‐established role in tissue repair and fibrosis by elevating collagen gene expression and fibroblast proliferation [114]. In the BLM‐induced mouse model, mangiferin significantly reduced TGF‐β1 levels and production of α‐SMA, which lowered the incidence of pulmonary fibrosis (Table 1). Furthermore, mangiferin treatment resulted in an elevation in E‐cadherin, an epithelial marker that is normally downregulated during EMT, suggesting a reversal of the EMT process in a study of the A549 alveolar epithelial cell line [32].

### 4.4 Potential Effect of Mangiferin Against Lung Pathogens

Mangiferin has drawn much attention because of its potential protective effects against various pathogenic diseases [115, 116, 117]. Recent studies found that mangiferin also has protective effects against respiratory infections. Moreover, mangiferin is found to be an antiviral drug against the H1N1 influenza virus in an *in silico* investigation [118]. A molecular docking investigation has demonstrated that mangiferin inhibits the H1N1 neuraminidase (NA) enzyme. This finding underscored its potential as an inhibitor of viral replication, indicating that it may be an effective anti‐viral drug for the treatment of influenza A H1N1 [118]. Mangiferin was also studied for its effects on inflammation and viral adsorption in human lung cells [119]. In addition, a recent study described the effect of mangiferin in lowering the symptoms of upper respiratory infections and stomachaches in healthy school‐age children. The study also indicated that children who took mangiferin had a lower risk of experiencing gastrointestinal illnesses such as diarrhea, vomiting, and other gastrointestinal issues. These results highlight the broad range of mangiferin's approach to treating respiratory infections, making it suitable for future preventative and therapeutic measures [120]. Recently, it has been reported that mangiferin in Qingjin Huatan decoction reduced influenza A virus pneumonia‐induced chemokines, their receptor‐related genes, and JAK2/STAT3 pathway in lung tissue in mice [121].

### 4.5 Effect of Mangiferin on Other Related Pathologies

In NSCLC cells treated with mangiferin, autophagy and extrinsic apoptosis are implicated in the weakening of LPS‐induced proliferation and the upregulation of LC3 expression [82]. Mangiferin upregulates E‐cadherin expression while downregulating vimentin in NSCLC cells. It also changes the way the PER1 gene is expressed. Mangiferin prevents LPS‐induced mitochondrial depolarization in NSCLC cells. In addition, it causes a substantial rise in caspase‐8, initiates apoptosis by extrinsic mechanisms, and stimulates autophagic LC3β/LC3α [82].

After mangiferin was administered in a BLM‐induced mice model, there was an apparent reduction in the levels of poly (ADP‐ribose) polymerase (PARP), NF‐κB p65, pro‐apoptotic protein Bid, and an increase in the levels of the anti‐apoptotic protein Bcl‐2 observed [31]. Mangiferin regulates the imbalance of Th1/Th2 cell differentiation by suppressing elevated levels of STAT6 and GATA‐3, which are linked to cytokine expression [72]. Mangiferin treatment suppresses the production of GATA‐3, ROR, and upregulates T‐bet [73]. Activation of NLRP3 and pyroptosis triggered by LPS is also inhibited by mangiferin treatment [75]. In the mice treated with mangiferin, the levels of IgG and IgM immunoglobulins were markedly increased, whereas the levels of IgA [76] and IgE were significantly decreased [29].

Mangiferin was found to lower the activity of NADPH‐cytochrome P450 reductase, which is responsible for transferring electrons to cytochrome P450 or cytochrome b5 during oxidative metabolism [122] as well as a decrease in NADPH and cytochrome C [77]. The mitochondrial pathway and PKC/NF‐κB pathway inhibition may be involved in mangiferin‐induced apoptosis in lung cancer cells [85].

The rate‐limiting enzyme in heme biosynthesis, heme oxygenase‐1 (HO‐1), has anti‐inflammatory, anti‐proliferative, anti‐apoptotic, and antioxidant properties [123]. Mangiferin also downregulates NF‐κB signaling and activates HO‐1/Nrf2 pathways [73]. Interestingly, in male albino rats with cigarette smoke‐induced chronic obstructive pulmonary disease, mangiferin restored the level of HO‐1 [30]. Moreover, mangiferin therapy has been shown to substantially decrease VCAM‐1 expression [80]. Mangiferin stimulated γ‐GCS and Nrf2 signaling [30] in addition, mangiferin downregulated caspase‐3, 8, 9, Bcl2, and Bax [81].

## 5 Effects of Mangiferin on Signal Transduction Pathways

### 5.1 Nrf2 Pathway

The Nrf2 pathway is associated with the anti‐inflammatory process by coordinating the recruitment of inflammatory cells and controlling gene expression via the antioxidant response element (ARE). Mangiferin activates the Nrf2 signaling pathway, facilitating its dissociation from Keap1, a substrate adapter for cullin‐based E3 ubiquitin ligase that prevents Nrf2 activation via ubiquitination and proteasomal destruction in normal conditions [124]. Nrf2 interacts with ARE and Maf protein to start gene expression that produces antioxidants and reduces oxidative stress markers, protecting lung cells from damage [73]. Furthermore, mangiferin lowers etoposide‐induced DNA damage in hematopoietic cells by activating the Nrf2/ARE pathways [125].

### 5.2 NF‐κB Pathway

The NF‐κB family plays a well‐established role in inflammation and innate immunity. Individuals with asthma and COPD exhibited elevated amounts of NF‐κB in their bronchial biopsies [126, 127]. Moreover, NF‐κB stimulates the NLRP3 inflammasome, resulting in caspase‐1 activation and the synthesis of IL‐1β and IL‐18, which cause inflammation and respiratory impairment [75]. In a mouse model, mangiferin exhibits considerable anti‐inflammatory properties by impeding NF‐κB activation in ovalbumin‐induced allergic rhinitis [73].

### 5.3 TGF‐β Pathway

Transforming growth factor‐β (TGF‐β) is a pleiotropic cytokine that is expressed by almost all cell types and tissues. TGF‐β signal transduction is essential for immunological homeostasis, tissue repair, and embryonic development [128]. In pathological conditions, excess TGF‐β results in extracellular matrix (ECM) deposition and EMT, which causes fibrotic disease [129]. Mangiferin reduced BLM‐induced inflammation and pulmonary fibrosis by inhibiting the TLR4/p65 pathway and the EMT process through interruption of the TGF‐β1/Smad2/3 pathway in mice [32]. Furthermore, it minimizes cardiac fibrosis in D‐galactose‐induced aging rats by interrupting the TGF‐β signaling pathway [130].

### 5.4 MAPK Pathway

Mitogen‐activated protein kinase (MAPK) cascades play a key role in mediating the conversion of extracellular signals into cellular responses. There are more than a dozen MAPK enzymes found in mammals that collectively regulate vital cellular functions, including proliferation, differentiation, motility, and survival [131]. Mangiferin protects against ischemia–reperfusion (IR) damage in streptozotocin‐induced diabetic rats by modulating the AGE‐RAGE/MAPK pathways, thus reducing oxidative stress, inflammation, and apoptosis in the myocardium [132]. In a male Wistar albino rat model, pretreatment with mangiferin 20 and 40 mg/kg significantly lowers cisplatin‐induced acute renal damage by altering the MAPK pathway [133].

### 5.5 Wnt/β‐Catenin Pathway

The growth and function of the hematopoietic system, hair follicle renewal, lung tissue repair and metabolism, liver metabolism and regeneration, and osteoblast maturation and activity are all associated with the Wnt signaling pathway [134, 135, 136]. Mangiferin protects against intestinal ischemia/reperfusion‐induced liver injury via the Wnt/β‐catenin pathway [137]. Again, it regulates the Wnt/β‐catenin signaling pathway by suppressing AKR1C3 and α‐synuclein accumulation in 6‐OHDA‐induced Parkinson's disease (PD) in C57BL/6 mice models. This activation restores β‐catenin levels, facilitating neuroprotection and improving the survival and functionality of dopaminergic neurons. In the in vitro context, mangiferin shielded cell damage caused by 6‐OHDA‐induced PD in the PC12 cell line of rat adrenal medulla pheochromocytoma through oxidative stress reduction, mitochondrial membrane potential restoration, and enhancement of tyrosine hydroxylase expression [138].

### 5.6 PI3K/AKT Pathway

A key intracellular signaling cascade that is essential in controlling the cell cycle is the PI3K/AKT pathway. It is intricately associated with processes including cancer formation, cell quiescence, and proliferation [139]. G protein‐coupled receptors, small GTPases, and receptor tyrosine kinases are some of the upstream signals that activate this pathway. When PI3K is activated, it facilitates the conversion of PIP2 to PIP3, which attracts PH‐domain‐containing membrane proteins including mTORC2 and AKT [140]. Mangiferin exerts a dose‐ and time‐dependent response to gastric cancer cells (SGC‐7901 and BCG‐823), preventing their proliferation and causing them to undergo apoptosis. It suppresses the PI3K/Akt/mTOR signaling pathway, upregulates pro‐apoptotic markers (Bax, Bad, cleaved caspase‐3), and downregulates anti‐apoptotic proteins (Bcl‐2, Bcl‐xL, and Mcl‐1) [141].

## 6 Limitations and Future Perspectives

The potential health benefits of mangiferin are limited by several factors. Mangiferin has a very low oral bioavailability, as evidenced by several studies [35, 142]. According to research on rats, the bioavailability can be 1.2% [35]. The low lipophilicity, limited intestinal membrane permeability, and low oral absorption of mangiferin could be the cause [143]. According to reports, mangiferin undergoes intense hepatic first‐pass metabolism, which reduces the amount of the dosage that enters the systemic circulation. This is one of the factors causing poor oral bioavailability in normal rats, which lowers the effectiveness [144]. Mangiferin showed a slower rate of elimination, a greater extent of distribution, and increased binding affinity when administered as a polyherbal formulation compared to its pure form [145]. Following systemic circulation, drug molecules may bind to plasma proteins. When small molecules are protein‐bonded, they cannot penetrate biological membranes and, therefore, cannot trigger a pharmacological response [146]. It may necessitate higher doses of mangiferin to achieve efficacious free concentrations. A pharmacokinetic study involving healthy male volunteers reported that after a single oral dose of 0.9 g of mangiferin, the peak plasma concentration reached was approximately 38.64 ± 6.75 ng/mL (about 0.09 μM) within 1 h, with an elimination half‐life of 7.85 ± 1.72 h [147]. When a significant dose of mangiferin was administered, its absorption increased. The findings suggest that achieving an effective concentration of mangiferin in human plasma through oral administration is challenging and limits the health‐promising effects [147]. Further, evaluating the potential use of mangiferin as a medicinal compound in humans requires extensive toxicity studies, which are currently limited. An acute toxicity test in rats found no significant toxic effects after dermal exposure to mangiferin at 2000 mg/kg, although some transient symptoms such as dyspnea occurred with oral administration. In the same study, 28‐day oral administration showed no altered hematology and clinical signs at doses up to 1000 mg/kg. However, histopathological changes including necrosis, vacuolar degeneration, and increment of apoptosis of the acinar cells in the exocrine pancreas were noted at 1000 mg/kg [36]. Additionally, mango leaf extract containing 60% mangiferin demonstrated no genotoxicity and established a no‐observed‐adverse‐effect level (NOAEL) of 2000 mg/kg in a 90‐day study in rats [148]. According to a subchronic toxicity study on Swiss albino mice at a dose of 200 mg/kg for 28 days, pure mangiferin and mangiferin solid dispersion (HPTR) did not result in any evident toxicity [149]. Detailed studies and clinical trials are required to investigate the bioavailability, pharmacokinetic, safety, and efficacy of mangiferin to develop therapeutics for humans.

Little is known about how specifically mangiferin may be modified, formulated, and used to treat respiratory disorders. Drugs for the treatment of respiratory diseases are often administered via inhalation [150]. In the inhalation drug delivery system, rapid delivery of an inhaled drug occurs over the large surface area of the respiratory tract epithelium. Bypassing first‐pass metabolism, drugs absorbed into the pulmonary circulation go straight via the pulmonary vein and into the systemic circulation [151]. As the inhalation delivery method targets the lungs directly, lower doses of the drugs are required for effective concentration, and fewer side effects are observed than with other systemic methods [152]. Delivering mangiferin through an inhalation delivery method could be a promising approach to overcoming dosing challenges for respiratory diseases. However, there are no well‐established methods or studies on developing effective inhalation delivery systems for mangiferin. Future studies should concentrate on developing effective mangiferin inhalation delivery methods.

There are several newly developed and potential formulation strategies to improve the bioavailability of mangiferin [37]. It has been shown that mangiferin can be encapsulated in nanoparticles, which serve as carriers to prevent degradation and make transport to the target cell easier. This technique is used to improve bioavailability, safety, and effectiveness [153]. To increase the bioavailability and water solubility of mangiferin, Zhou et al. generated transferrin‐modified mangiferin‐loaded solid lipid nanoparticles (Tf‐modified MGF‐SLNs) and demonstrated that Tf‐MGF‐SLNs were effective in suppressing tumor growth, suggesting potential applications of such formulations in lung cancer [154]. Moreover, when paired with vitamin E TPGS (D‐α‐Tocopheryl Polyethylene Glycol 1000 Succinate), a specialized nano‐micelle system (SPNMS), the solubility, bioavailability, and anti‐cancer efficacy of mangiferin were increased. Optimized micelles (< 60 nm) exhibited threefold improved bioavailability in rats, better lymphatic absorption, increased breast cancer cell uptake, and over 80% drug release in 15 min [155]. Furthermore, inclusion complexes with cyclodextrins, such as β‐cyclodextrin, may improve the stability and water solubility of mangiferin, which potentially enhances its bioavailability [156].

## 7 Conclusion

The current review demonstrates that mangiferin mediates various protective effects such as antioxidant, anti‐inflammatory, analgesic, and immunomodulatory effects. These results highlight its potential utility in preserving a healthy ratio of ROS to antioxidants by lowering intracellular ROS levels. According to some research, mangiferin increases antioxidant activities in the respiratory tissues of mice and rats, which in turn stabilizes the actions of superoxide dismutase. This improves the oxidative defense in the animal lungs. Furthermore, it has the potential to impact lung cell physiology by lowering the levels of respiratory ROS and intracellular MDA, as well as acting as an efficient ROS scavenger. The findings of this investigation provide additional evidence supporting the potential therapeutic application of mangiferin in the management of allergic rhinitis by activating Nrf2/HO‐1 signaling and inhibiting NF‐κB signaling. In allergic asthma, inflammatory cytokines from Th1 and Th2 contribute to disease pathogenesis. Mangiferin treatment markedly attenuated various cytokines. The review also highlighted that mangiferin reduced TGF‐β1 levels, which plays a vital role in tissue fibrosis (see Figure 4). The broad impact of mangiferin presents a potentially valuable therapeutic agent for diseases related to the respiratory tract. In addition, mangiferin exhibits certain limitations. Although several studies have reported a lack of significant toxicity in rats, the low bioavailability of mangiferin due to its limited absorption from the gastrointestinal tract is associated with adverse effects such as diarrhea and nausea in humans. The efficacy of this therapeutic agent may be impeded due to the necessity of administering higher doses to attain the intended therapeutic outcomes. Several studies have reported the lack of significant toxicity associated with mangiferin. Due to its structural composition, the compound exhibits a proficient ability to scavenge free radicals. A further constraint pertains to the absence of exhaustive clinical trials that precisely assess the effectiveness of mangiferin in combating respiratory tract infections. Although numerous preclinical investigations have showcased mangiferin's antimicrobial and anti‐inflammatory characteristics, additional research is necessary to substantiate these outcomes in human participants.

For pulmonary diseases, alternative delivery methods such as inhalation may provide a feasible strategy to overcome dosing challenges by ensuring localized drug delivery and minimizing systemic exposure. Moreover, advances in formulation methodologies such as prodrug design, nanoencapsulation, or chemical modifications could significantly enhance mangiferin's bioavailability and stability, enabling its broader therapeutic application. Synergistic approaches also hold promise, as combining mangiferin with antibiotics or other natural antimicrobial agents has demonstrated enhanced efficacy. Such combinations could reduce the required dosage of conventional antibiotics, minimize adverse effects, and potentially address antimicrobial resistance. These strategies, combined with robust clinical trials, are essential for validating mangiferin's efficacy and safety as a treatment for respiratory diseases.

While challenges remain, the potential of mangiferin as a therapeutic agent for respiratory tract infections is remarkable. Future research should address the pharmacokinetic and formulation challenges, explore innovative delivery systems, and validate their therapeutic effects through well‐designed clinical studies.

## Author Contributions

**Kazi Ahsan Ahmed:** data curation, investigation, validation, writing – original draft, writing – review and editing. **Nusrat Afrin:** investigation, software, visualization, writing – original draft, writing – review and editing. **Popy Ghosh:** investigation, writing – original draft, writing – review and editing. **Irin Amin Heya:** writing – original draft. **Sajidur Rahman Akash:** writing – original draft, writing – review and editing. **Akhi Moni:** writing – review and editing. **Mohammad Nazrul Islam:** writing – review and editing. **Md. Golzar Hossain:** writing – review and editing. **Alessandra Sinopoli:** resources, writing – review and editing. **Md Abdul Hannan:** validation, visualization, writing – review and editing. **Md Jamal Uddin:** conceptualization, methodology, project administration, software, supervision.

## Conflicts of Interest

The authors declare no conflicts of interest.

## Data Availability

Data sharing is not applicable to this manuscript since this study did not use or analyze any new data.
